# Supplementary material for: Genome-Wide Association Studies of Key Traits in Apis cerana cerana (Hymenoptera: Apidae) from Guizhou Province
Source: Genes (Basel). 2025 Sep 27;16(10):1148. doi: 10.3390/genes16101148 (PMC12562913; doi:10.3390/genes16101148)
Supplement: Supplementary file 1 [file genes-16-01148-s001.zip › genes-3856827-supplementary.pdf]

Supplementary Table S1. SNP information of *Apis cerana cerana* in 12 regions of Guizhou Province.

Traits	Chromosome	Position	Nucleotide Variation	-log10(*p*-value)
TIII & IVL	KZ288189.1	1 126 463	G→A	6.22
TIII & IVL	KZ288192.1	2 461 641	A→G	7.95
TIII & IVL	KZ288193.1	1 630 986	C→G	6.38
TIII & IVL	KZ288194.1	415 535	A→G	6.82
TIII & IVL	KZ288205.1	142 314	T→C	6.90
TIII & IVL	KZ288213.1	179 195	G→A	6.03
TIII & IVL	KZ288220.1	3 956	C→T	10.13
TIII & IVL	KZ288227.1	3 276 796	T→C	6.32
TIII & IVL	KZ288227.1	3 276 797	T→C	6.32
TIII & IVL	KZ288229.1	819 881	C→T	6.19
TIII & IVL	KZ288255.1	1 116 041	T→A	6.45
TIII & IVL	KZ288255.1	1 118 450	G→A	6.50
WML3	KZ288192.1	706 123	A→G	6.67
WML3	KZ288192.1	706 219	G→A	6.18
WMI3	KZ288185.1	1 939 427	G→A	6.01
WMI3	KZ288227.1	1 866 677	C→T	6.09

Notes: TIII & IVL: tergite III & IV length; WML3: wax mirror length on sternite III; WMI3: wax mirror interval on sternite III.

Supplementary Table S2. SNPs with a significant genome-wide association with traits.

Traits	Gene ID	Functional annotation
TIII & IVL	*APICC_05611*	Longitudinals
TIII & IVL	*APICC_06479*	Longitudinals
TIII & IVL	*APICC_06480*	Zinc
TIII & IVL	*APICC_02050*	Lamin
TIII & IVL	*APICC_05706*	Leishmanolysin
TIII & IVL	*APICC_02236*	Radial
TIII & IVL	*APICC_05419*	Hypothetical
TIII & IVL	*APICC_07473*	BTB/POZ
TIII & IVL	*APICC_07474*	ATP-dependent
TIII & IVL	*APICC_09312*	Nurim
TIII & IVL	*APICC_09560*	Dynein
WML3	*APICC_03144*	Protein
WML3	*APICC_08157*	Hypothetical
WMI3	*APICC_00038*	Protein
WMI3	*APICC_00039*	Hypothetical
WMI3	*APICC_06559*	Hypothetical
WMI3	*APICC_06952*	Hypothetical
WMI3	*APICC_08164*	Cytosolic
WMI3	*APICC_08492*	Hypothetical
WMI3	*APICC_09369*	Phospholipase

Notes: TIII & IVL: tergite III & IV length; WML3: wax mirror length on sternite III; WMI3: wax mirror interval on sternite III.
